# Early care in childhood and psychological burden among East and West German adults

**DOI:** 10.1186/s12889-026-26398-1

**Published:** 2026-02-06

**Authors:** L. Kriechel, M. Beutel, V. Clemens, E. Brähler

**Affiliations:** 1 Department of Psychosomatic Medicine and Psychotherapy, University Medical Center of the Johannes Gutenberg University Mainz, Untere Zahlbacher Str. 8, Mainz, 55131 Germany; 2 Federal Institute for Population Research, Friedrich-Ebert-Allee 4, Wiesbaden, 65185 Germany; 3 Department for Child and Adolescent Psychiatry/Psychotherapy, University of Ulm, Steinhövelstr. 5, Ulm, 89075 Germany; 4 Department of Medical Psychology and Medical Sociology, University of Leipzig Medical Center, Philipp-Rosenthal-Str. 55, Leipzig, 04103 Germany

**Keywords:** External childcare, Familial childcare, Psychological burden, East/West germany

## Abstract

**Background:**

The consequences of external childcare for children are controversially discussed. Many claim that early extrafamilial care is harmful to the child. This article aimed to study the relationship between external childcare at preschool age and psychological burden in adulthood. Given that extrafamilial care followed different norms and regulations depending on the location in East or West Germany during their division, the question was also pursued whether the association between early childcare and psychological burden differed between those regions.

**Methods:**

The analyses are based on a representative sample collected in 2020. A total of 1,796 Germans (1,448 West, 348 East) were divided into three childcare groups: those who first entered external care before the age of three, those who started with or after the age of three, and those who stayed in familial care until school entry. Psychological burden was indicated by the Brief Symptom Inventory-18. Differences in psychological burden according to childcare group and region were tested by ANOVAs, ANCOVAs, and OLS-regressions.

**Results:**

Compared to West Germans in familial care before school entry, West Germans who received full-time external childcare before the age of three tended to report stronger symptoms of depression (std. β = 0.20, *p* = .050), anxiety (std. β = 0.20, *p* = .056), and the global score of psychological burden (std. β = 0.19, *p* = .066). In contrast, extrafamilial childcare was not related to the psychological burden of East Germans. Moreover, East Germans and West Germans differed significantly in terms of their relationship between psychological burden and extrafamilial full-time care before the age of three.

**Conclusions:**

Though in the West, full-time care before the age of three was related to greater psychological burden this was not found in the East, indicating external childcare itself might not be harmful. Future research should observe whether selection effects, differing quality in childcare institutions, or differing norms were responsible for this disparity between regions.

## Background

For decades, it has been debated how children should be cared for, especially before school entry. In the case of Germany, two divergent approaches to childcare have manifested. Because of the historically disparate laws, labor structures, and social benefits during the German division, differing attitudes and behaviors regarding childcare have arisen in the German Democratic Republic (GDR) and the Federal Republic of Germany (FRG). While the GDR promoted external childcare and requested mothers to be fully integrated into the labor market [1], West German families were incentivized if mothers took care of their preschoolers [2]. In 1988, 80% of East German children and 3% of West German children younger than three years of age attended a crèche, while 94% of East German children and 60% of West German children older than two years had entered kindergarten [2]. With these shares, the differing institutionalized norms regarding childcare become even more apparent. Entering an external childcare facility below the age of three was also difficult in West Germany because of lacking opportunities; the chances were higher in cities [3]. Moreover, employed, more educated, and single mothers were more likely to rely on external childcare for their children below the age of three [3].

Early external care in the GDR elicited criticism. Weber (1996) [4] cd the GDR’s childcare systems one of their greatest achievements, whereas others claimed a prevalent lack of love for children and governmental malinvestments. Generally, Israel (2008) assumes that the GDR’s caregiving had negative consequences for children, as she claims that children were separated from their parents too early. Moreover, the prevalence of insecure child-caretaker relations in the GDR was high – this might have derived from the fact that East German caretakers were taught to focus on peer interactions instead of their attachment to children [6]. Similarly, it was stated that while emotional care was neglected in crèches, kindergartens lacked latitude for individual development [5].

Even today, East and West Germany differ: while in the West 90.4% of children between the ages of three and six and 32.7% below the age of three have attended external care in 2023, the respective shares for the East are 93.3% and 54.2% [7]. The emotional debate on the adequate caretaking of children is also ongoing; it is argued that children are idealized and romanticized which is why parents neglect their own well-being to serve their children’s needs [8, 9]. Again, this is mostly prevalent in West Germany. A study showed that West Germans deem fathers and grandparents the second and third best caretakers after the mother of small children, whereas East Germans consider the crèche and then fathers the second and third best options [10].

As extrafamilial care is becoming more prevalent, research on risk and protective factors related to the association between childcare and child well-being has increased. For example, the bond between an employed mother and her child is more secure if the mother exhibited less anxiety about external childcare and was more committed to her work before giving birth [11, 12]. Since East German women are more likely to consider external care the second best option regarding caretaking, their approval of it along with their internalized commitment to work [13] indicate that their mother-child bond benefits from this situation. In contrast, in 1993, West German women were the least inclined to engage in paid labor [13]. It was also found that congruence between gender norms on childcare and the employment status of mothers could be confirmed for both GDR and FRG [14]. This again suggests that West German mothers should have been more afraid of external care than East Germans during times of division.

An extensive amount of previous research has observed the outcomes of several forms of childcare for children until adolescence. Upon the first entry into external care, children experience separation anxiety [15], but after a few months, the distress levels of all children decrease [16]. This can partially explain why there is no difference in attachment style between children who attended external care below the age of 15 months and those who did not [17, 18]. While a meta-analysis revealed that the distress levels of children in external care were greater than those in familial care [19], Finnish children in external care exhibit 30% lower distress levels than those in familial care, presumably due to the former’s more consistent daily rhythms [20]. The country-specific context or quality of childcare institutions might explain this discrepancy. However, research found that externalizing behavior of kindergarteners was associated with time they had spent in external care at a younger age [21]. Also the age of entry into external care matters: Externalizing behavior was least pronounced among those who entered external care between the ages of two and three [22]. Externalizing behavior in childhood is furthermore related to a range of mental disorders in adulthood, such as anxiety and mood disorders [23]. This paper aims at adding knowledge to the gap of research on the relationship between early childcare and adult psychological outcomes. Rarely, studies use the retrospective information on respondents’ early childcare situation to predict their current state. This is why little research on external care and its characteristics in adulthood could be found. One study compared anxiety and depression scores between 162 university students in two German cities; these scores did not differ between childcare groups or between East and West Germans [24]. Moreover, psychological distress, physical health, anxiety, and physical complaints in adulthood do not vary between East Germans who attended a crèche and those who did not [25]. Among those who were born and socialized in the GDR, even a more distinct differentiation of childcare into first external care before the age of three or older does not yield significant disparities regarding depression, anxiety, or somatization in adulthood [26]. Furthermore, the health behavior of adults who went to preschool is partially better than that of adults who did not attend preschool: The former group is less prone to smoking or multiple behavioral risk factors, but is more likely to participate in sports or consume vegetables [27, 28]. The better health of preschool attendees in adulthood may be explained by high quality kindergarten education profiting cognition which leads to greater attainment and thus a better job; the consequential higher income and fewer occupational hazards lead to reduced psychological distress [29]. This signals that the potential association between childcare and later health in adulthood can most probably be explained by path dependency. The mechanisms that underly the paths between early childhood care and later health outcomes are potentially multifaceted and can be related to education, the socio-economic situation during formative years, one’s disposition, behavior, or other influences.

The preceding paragraphs showed that both positive and negative outcomes of external childcare at preschool age were found. Furthermore, only one study could be found that compared the association between mental health in adulthood and external childcare among East Germans and West Germans, using a rather small and homogeneous sample. This is why this article aims to add to the discussion on external childcare by providing a more distinct specification of the form of external childcare and observing psychological burden in adulthood. Oftentimes, researchers compare only preschoolers to nonpreschoolers. In contrast, the data used in this article enabled us to distinguish the age of first entrance into a childcare facility. Moreover, the integration of socialization in East or West Germany also offered the possibility of indicating diverging childcare norms at the time of the respondents’ childhoods. Therefore, the questions asked are whether early external childcare is associated with psychological burden in adulthood and whether there are regional differences between East and West Germany. As previous research has found with a larger sample size but with other constructs for psychological burden that psychological burden is not related to early childcare of East Germans [26], this manuscript first replicates this finding. Moreover, it enabled us to interpret the findings for the West German population.

## Methods

### Sample

A representative survey of the German general population, including 2,503 respondents, was collected by a commercial survey institute (Independent Service for Survey, Methods and Analysis [USUMA]) from February until April 2020. The study was approved by the Ethics Committee of the Medical Department of the University of Leipzig (002/20-ek) and was conducted in accordance with the 1964 Declaration of Helsinki and its later amendments. All participants gave their informed consent prior to their response. A multistage random-route technique was used, in which sample points were first selected, followed by a random route procedure to randomly choose households. Participants within the chosen households were targeted using a kish-selection grid of household members who were at least 14 years old and were able to speak German. The response rate was 44.1%, mostly due to refusal to identify a participant, failure to contact respondents, or refusal to participate.

Some respondents were excluded from the analyses within this article. Only participants who were raised predominantly in Germany and born in 1949, when the FRG and the GDR adopted their basic law and constitution, or after were included. In total, *n* = 315 participants were excluded because they were born before 1949. Since there were no statistical differences between those born before and after unification, no upper birth year limit was set. Participants who stated that at preschool age, they entered external childcare facilities by the week with overnight stays, seasonally, or on a long-term basis were omitted due to their small sample size (*n* = 26 were excluded). Only respondents who still lived in the region (East/West) of their upbringing were included (*n* = 257 were excluded) to prevent the region of living from confounding the impact of the region respondents where have been socialized. Finally, *n* = 109 respondents with missing values for any of the variables used were excluded, though *n* = 96 of them missed relevant information on their first external childcare, which is why a listwise deletion was suitable. Thus, the final sample consisted of *N* = 1,796 respondents.

### Variables

Region in East or West Germany: To determine if respondents were predominantly raised in former Eastern or Western Germany, they were asked: “In which German federal state did you predominantly grow up?”. The German state respondents lived in at the time of the survey was recorded, as well. Respondents who were predominantly raised and still living in the West were coded as West Germans (0), while those who grew up and were still living in the East were labelled East Germans (1).

#### First external childcare

The question “How were you cared for at a preschool age?“ was divided into the subsequent subquestions “before the age of three predominantly by […]” and “between the age of three until school enrollment predominantly by […]“, to determine the respondents’ age at first external childcare admission. Allowing for multiple selection, “mother”, “father”, “other relatives”, “daycare”, “foster home”, “crèche” (alternatively between the ages of three and six “kindergarten”), “children’s home” and “other” could be selected. If the participants chose “daycare”, “crèche”/“kindergarten” or “children’s home”, they were asked to specify the duration of their external caretaking (“part time”, “full time”, “seasonal”, “weekdays with overnight stays” or “permanently”). Due to the small sample size of West Germans in full-time care younger than the age of three and East Germans, in general, the additional stratification of daily time spent in external care was relinquished. Therefore, three childcare groups could be formed for analyses, differing between the first age of extrafamilial childcare: a) familial care, as respondents did not respond with either “daycare” or “crèche”/“kindergarten”; b) external daycare below the age of three; and c) external daycare between the age of three and school enrollment.

#### Psychological burden

To measure the respondents’ psychological burden, the validated German version of the Brief Symptom Inventory (BSI-18; [30, 31]) was used. The BSI-18 is a short form of the Symptom-Checklist 90-R. The construct was validated for students, a non-clinical sample, and a clinical sample [32]. It respectively comprised six variables indicating depression, anxiety, and somatization. Using these items, three sum scores ranging between 0 and 24 were calculated with low values indicating less psychological burden and high values indicating a high psychological burden. Additionally, estimating a sum score of each item, the Global Severity Index (GSI) exhibits a measure of the respondents’ general psychological burden varying between 0 and 72.

#### Control variables

As adverse childhood experiences are a risk factor for psychological burden in adulthood [33, 34], they were integrated as a control variable. Furthermore, by integrating them, mean values for each childcare group can be observed to indicate whether a certain childcare group was associated with a pathological household. The German version of the Adverse Childhood Experiences (ACE) questionnaire [35] contains ten questions on experiences with emotional, physical, and sexual abuse, as well as emotional and physical neglect, separation from one of the parents, violence against the mother, a household member’s substance abuse, mentally ill or suicidal household members, and finally incarceration of a household member before the age of 18 [36]. The respondents could use a dichotomous scale to indicate “yes” or “no” whether they experienced the described incidences. A sum score was then built from the number of experiences that were answered with “yes” (range 0–10). The dummy variables of sex (male/female) and university entrance degree (no/yes) were additional control variables. Age was implemented as a continuous measure. Household income was assessed with a categorical scale with 13 categories. Using the ranges’ averages, household income could be transformed into a quasimetric variable. For a better interpretation, the variable was then divided by 1,000. Finally, since respondents were surveyed before and during the first lockdown due to the COVID-19 pandemic, the month of the interview was controlled for, as well.

### Analyses

All analyses were performed with R (version *2023.12.1 + 402*). First, descriptive group comparisons were estimated between East and West Germans as well as between childcare groups using post-hoc Tukey HSD (honestly significant difference) tests and post-hoc Chi-Square tests. Second, linear regressions were conducted to predict the outcome variables, separately for East and West Germans to distinctly explore the associations between all predictors and psychological burden. Third, using tests of covariance (ANCOVA), the differences between the regression intercepts of East and West Germans were tested for the childcare groups.

## Results

### Descriptive results

Tables 1 and 2 show the descriptive results for East and West Germans. Group comparisons between region and form of early childcare were included. Compared to West Germans, East Germans exhibited lower values of depression (W: 1.63 vs. E: 0.85, *p* <.001), anxiety (W: 1.41 vs. E: 0.59, *p* <.001), somatization (W: 1.27 vs. E: 0.37, *p* <.001), and the global scale (W: 4.32 vs. E: 1.77, *p* <.001), indicating a lower psychological burden. This corresponds to previous research during the last few years [37, 38]. Moreover, they reported adverse childhood experiences less frequently than West Germans did (W: 1.16 vs. E: 0.40, *p* <.001) which also relates to previous findings [39]. Additionally, East Germans had a lower household income (W: 2,734.51 vs. E: 2,48.56, *p* <.001) than West Germans. Furthermore, East and West differed significantly regarding each childcare group (*p* <.001), suggesting statistically different regional shares for each of them. In the East, with a share of 43.97%, most respondents entered external care before the age of three, followed by the 30.46% who entered external care between the ages of three and six. In total, 25.57% of East Germans were only in familial care at preschool age. In contrast, West Germans predominantly entered external care between the ages of three and six with a share of 47.17%, shortly followed by 46.96% who were only in familial care. Only 5.87% of West German respondents entered external care before the age of three.

**Table 1:** Descriptive results for East Germans in total and per childcare group

Form of first early childcare	Total	a. only familial (*n* = 89; 25.57%)	b. < 3 (*n* = 153; 43.97%)	c. 3–6 (*n* = 106; 30.46%)
	N (%)/M (SD)	N (%)/M (SD)	N (%)/M (SD)	N (%)/M (SD)
Depression	0.81^***^ (1.63)	0.82 (2.05)	0.80 (1.57)	0.81 (1.32)
Anxiety	0.59^***^ (1.16)	0.45 (1.22)	0.63 (1.13)	0.65 (1.15)
Somatization	0.37^***^ (1.18)	0.40 (1.15)	0.18^c^ (0.62)	0.62^b^ (1.69)
GSI	1.77^***^ (3.23)	1.67 (3.9)	1.61 (2.52)	2.08 (3.51)
ACE	0.40^***^ (0.93)	0.30 (0.76)	0.43 (0.99)	0.42 (0.98)
Age	46.99 (15.96)	55.54^bc^ (12.68)	42.20^ac^ (14.79)	46.73^ab^ (17.16)
Female	145 (41.67)	34 (38.2)	65 (42.48)	46 (43.4)
University entrance degree	283 (18.68)	7^*^ (7.87)	38 (24.84)	20 (18.87)
In relationship	243 (69.83)	70 (78.65)	103 (67.32)	70 (66.04)
Household income	2,448.56^***^ (1015.75)	2,321.01 (824.17)	2,619.05^c^ (1147.32)	2,309.58^b^ (926.94)
Month of survey				
February	99 (28.45)	8 (8.99)^*^	49 (32.03)	42 (39.62)^*^
March	171 (49.14)	50 (56.18)	75 (49.02)	46 (43.40)
April	78 (22.41)	31 (34.83)^*^	29 (18.95)	18 (16.98)

The results for 348 East Germans are presented. The childcare groups a, b, and c depict the form of the respondents’ first external care. Thus, each respondent was assigned to only one group. Stars indicate the results of the Tukey HSD post-hoc tests regarding significant differences to West German respondents in the Total column and Χ^2^-test differences regarding the binary variables in columns a to c (^*^ *p* <.05 ^**^ *p* <.01 ^***^ *p* <.001). Superscript letters indicate the results of the Tukey HSD post-hoc tests for significant differences between the childcare groups

*GSI* Global Severity Index, *ACE* Adverse Childhood Experiences

**Table 2:** Descriptive results for West Germans in total and per childcare group

Form of early childcare	Total	a. only familial (*n* = 680; 46.96%)	b. < 3 (*n* = 85; 5.87%)	c. 3–6 (*n* = 683; 47.17%)
	N (%)/M (SD)	N (%)/M (SD)	N (%)/M (SD)	N (%)/M (SD)
Depression	1.63^***^ (3.19)	1.75 (3.39)	2.51^c^ (4.31)	1.40^b^ (2.79)
Anxiety	1.41^***^ (2.58)	1.52 (2.67)	2.07^c^ (4)	1.22^b^ (2.22)
Somatization	1.27^***^ (2.42)	1.43^c^ (2.54)	1.49 (3.45)	1.09^a^ (2.12)
GSI	4.32^***^ (7.26)	4.70^c^ (7.62)	6.07^c^ (11.18)	3.71^ab^ (6.15)
ACE	1.16^***^ (1.81)	1.22 (1.82)	1.36 (1.88)	1.07 (1.79)
Age	45.84 (14.61)	49.36^bc^ (14.67)	39.02^ac^ (13.69)	43.17^ab^ (13.79)
Female	750 (51.8)	346 (50.88)	44 (51.76)	360 (52.71)
University entrance degree	385 (26.59)	164 (24.12)	32 (37.65)	189 (27.67)
In relationship	906 (62.57)	419 (61.62)	40^*^ (47.06)	447 (65.45)
Household income	2,734.51^***^ (1210.24)	2,671.12 (1217.33)	2,583.59 (1217.06)	2,816.41 (1198.43)
Month of survey				
February	274 (18.92)	135 (19.85)	22 (25.88)	117 (17.13)
March	940 (64.92)	448 (65.88)	49 (57.65)	443 (64.86)
April	234 (16.16)	97 (14.26)	14 (16.47)	123 (18.01)

The results for 1,448 West Germans are presented. The childcare groups a, b, and c depict the form of the respondents’ first external care. Thus, each respondent was assigned only one group. Stars indicate the results of the Tukey HSD post-hoc tests regarding significant differences to East German respondents in the Total column and Χ^2^-test differences regarding the binary variables in columns a to c (^*^p < 05 ^**^p <.01 ^***^p <.001). Superscript letters indicate the results of the Tukey HSD post-hoc tests for significant differences between the childcare groups

*GSI* Global Severity Index, *ACE* Adverse Childhood Experiences

A descriptive comparison of the forms of first-time external childcare at preschool age is shown in Table 1. East Germans who were receiving full-time external childcare before the age of three were the group with the lowest psychological burden, as indicated by the GSI. However, the only significant difference among East Germans regarding psychological burden was given in the case of somatization by those who first entered external care before the age of three (c: 0.18), as they differed from those who entered external care between the ages of three and six (a: 0.62, *p* <.01). Furthermore, the three East German childcare groups differed from one another regarding their mean ages, with those in familial care being the oldest group and the youngest consisting of those who entered external care before the age of three. Those in the latter group also exhibited a significantly greater household income than did those who entered external care between the ages of three and six.

In contrast to East Germans, West Germans who entered external care before the age of three reached the highest GSI levels, suggesting the highest psychological burden. However, the descriptive results did not account for control variables or the rather small number of West German participants (*n* = 85) who entered external care before the age of three. This group differed from those who entered external care between the ages of three and six years regarding depression (b: 2.51 vs. c: 1.40, *p* <.01), anxiety (b: 2.07 vs. c: 1.22, *p* <.01), and the GSI (b: 6.07 vs. c: 3.71, *p* <.05). Moreover, West Germans who entered external care between the ages of three and six reached more beneficial somatization scores than did those in familial care (a: 1.43 vs. c: 1.09, *p* <.05). Again, all childcare groups differed regarding their age, with those in familial care being the oldest group and those who entered external care before the age of three being the youngest group. Furthermore, the latter were also significantly less likely to live with a partner within one household.

### Regression results

**Fig. 1:**
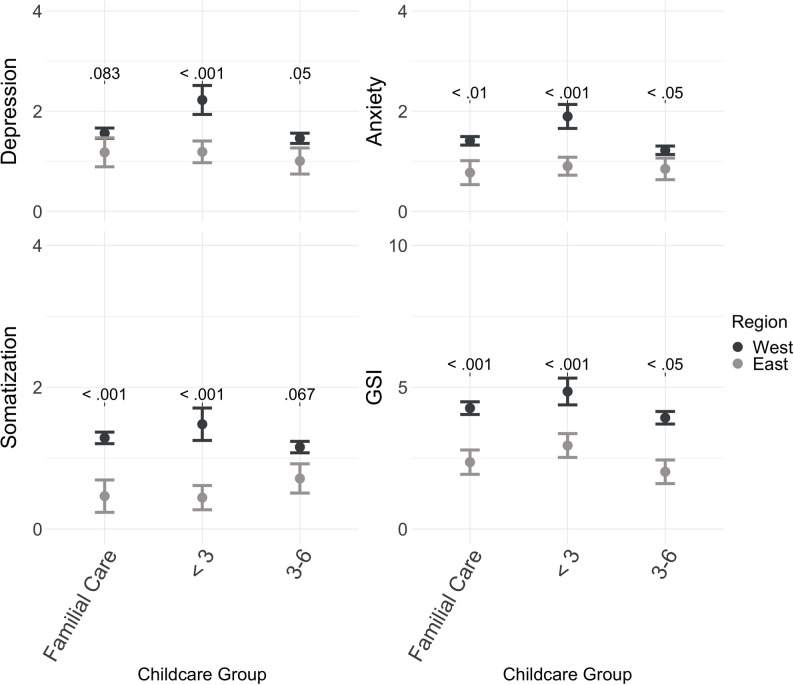
Adjusted means of the BSI-18 while differentiating between East and West and the childcare group. The adjusted means of the childcare group predictor for each dependent variable are portrayed on the y-axis. Significance levels were estimated by ANCOVAs to test for group differences between regions. The analyses controlled for adverse childhood experiences, age, sex, university entrance degree, partnership status, and household income. NS = not significant, GSI = Global Severity Index

Figure 1 illustrates the regression results of the models predicting psychological burden separately for East Germans and West Germans. The significance levels of the group differences between East and West Germans determined via ANCOVA are shown in Fig. 1. Furthermore, as presented in Fig. 1, it becomes more apparent that the association between psychological burden and external care before the age of three was stronger among West Germans than among Easterners, as the ANCOVA results predicted that each indicator of psychological burden differed between East and West (*p* <.001).

This was also highlighted when examining the distinct regression results for East and West Germans, as portrayed in Tables 3 and 4. Among Westerners, depression (std. β = 0.20, *p* =.050), anxiety (std. β = 0.20, *p* =.056), and the GSI (std. β = 0.19, *p* =.066) of those who entered external care before the age of three tended to differ from those of the reference group in familial care. In contrast, differences among East Germans were not significant and standardized β levels were lower.

**Table 3:** OLS-regression predicting the psychological burden in East Germany

	Depression			Anxiety			Somatization			GSI		
*Predictors*	*std. Beta*	*std. CI*	*p*	*std. Beta*	*std. CI*	*p*	*std. Beta*	*std. CI*	*p*	*std. Beta*	*std. CI*	*p*
(Intercept)	0.07	− 0.29–0.43	0.572	0.25	− 0.13–0.63	0.256	0.18	− 0.20–0.56	0.756	0.19	− 0.18–0.56	0.558
Age at first entry into external care (ref = only familial care at preschool age)												
External care aged < 3	0.13	− 0.14–0.39	0.343	0.02	− 0.26–0.31	0.875	− 0.04	− 0.32–0.24	0.763	0.06	− 0.22–0.33	0.681
External care aged 3–6	0.04	− 0.24–0.31	0.786	0.07	− 0.22–0.37	0.629	0.23	− 0.06–0.52	0.118	0.13	− 0.15–0.41	0.367
Control Variables												
ACE	0.30	0.20–0.40	**< 0.001**	0.21	0.10–0.31	**< 0.001**	0.10	− 0.00–0.20	0.062	0.27	0.16–0.37	**< 0.001**
Age	0.20	0.10–0.31	**< 0.001**	0.00	− 0.12–0.12	0.991	0.23	0.11–0.34	**< 0.001**	0.19	0.08–0.30	**0.001**
Female	0.06	− 0.13–0.26	0.520	0.13	− 0.08–0.34	0.214	− 0.07	− 0.28–0.14	0.516	0.06	− 0.15–0.26	0.590
University entrance degree	0.11	− 0.15–0.38	0.406	0.02	− 0.27–0.31	0.898	− 0.06	− 0.35–0.22	0.660	0.04	− 0.24–0.32	0.771
In relationship	− 0.26	− 0.52 – − 0.01	**0.041**	− 0.37	− 0.65 – − 0.10	**0.007**	0.00	− 0.27–0.27	0.997	− 0.27	− 0.53 – − 0.01	**0.044**
Household income	− 0.13	− 0.25 – − 0.01	**0.028**	0.14	0.01–0.26	**0.034**	− 0.05	− 0.18–0.07	0.404	− 0.04	− 0.16–0.08	0.549
Survey Month (ref = February)												
March	− 0.08	− 0.32–0.16	0.502	− 0.16	− 0.41–0.09	0.210	− 0.29	− 0.54 – − 0.04	**0.022**	− 0.21	− 0.45–0.04	0.097
April	0.17	− 0.11–0.46	0.224	0.00	− 0.30–0.31	0.976	− 0.22	− 0.52–0.08	0.143	0.01	− 0.28–0.30	0.953
R^2^/R^2^ adjusted	0.215/0.192			0.098/0.071			0.123/0.097			0.166/0.141		

A total of 348 East German respondents were included in the analyses. The results of four different OLS-regressions are shown. Significant *p* values (< .05) are printed in bold

*std*. standardized, *CI* 95% confidence interval, *GSI* Global Severity Index, *ACE* Adverse childhood experiences

**Table 4:** OLS-Regression predicting the psychological burden in West Germany

	Depression			Anxiety			Somatization			GSI		
*Predictors*	*std. Beta*	*std. CI*	*p*	*std. Beta*	*std. CI*	*p*	*std. Beta*	*std. CI*	*p*	*std. Beta*	*std. CI*	*p*
(Intercept)	0.15	0.00–0.29	**< 0.001**	− 0.06	− 0.21–0.08	**0.001**	− 0.00	− 0.15–0.15	0.215	0.04	− 0.10–0.18	**< 0.001**
Age at first entry into external care (ref = only familial care at preschool age)												
External care aged < 3	0.20	− 0.00–0.41	0.050	0.20	− 0.01–0.41	0.056	0.09	− 0.13–0.30	0.428	0.19	− 0.01–0.39	0.066
External care aged 3–6	− 0.03	− 0.13–0.07	0.549	− 0.06	− 0.16–0.04	0.219	− 0.05	− 0.15–0.06	0.376	− 0.05	− 0.15–0.05	0.306
Control Variables												
ACE	0.38	0.34–0.43	**< 0.001**	0.37	0.32–0.41	**< 0.001**	0.30	0.25–0.35	**< 0.001**	0.40	0.35–0.45	**< 0.001**
Age	0.06	0.01–0.11	**0.019**	0.03	− 0.02–0.08	0.176	0.14	0.09–0.19	**< 0.001**	0.08	0.04–0.13	**0.001**
Female	0.03	− 0.07–0.12	0.571	0.11	0.02–0.21	**0.019**	0.13	0.03–0.22	**0.010**	0.09	0.00–0.19	**0.044**
University entrance degree	0.09	− 0.02–0.20	0.104	0.09	− 0.02–0.20	0.098	0.08	− 0.03–0.19	0.170	0.10	− 0.01–0.20	0.072
In relationship	− 0.07	− 0.18–0.04	0.225	0.17	0.06–0.29	**0.003**	0.10	− 0.02–0.21	0.093	0.06	− 0.05–0.17	0.251
Household income	− 0.15	− 0.21 – − 0.10	**< 0.001**	− 0.12	− 0.18 – − 0.06	**< 0.001**	− 0.12	− 0.18 – − 0.06	**< 0.001**	− 0.15	− 0.20 – − 0.10	**< 0.001**
Survey Month (ref = February)												
March	− 0.16	− 0.28 – − 0.04	**0.008**	− 0.13	− 0.25 – − 0.00	**0.043**	− 0.16	− 0.28 – − 0.03	**0.014**	− 0.17	− 0.29 – − 0.05	**0.006**
April	− 0.21	− 0.36 – − 0.05	**0.010**	− 0.18	− 0.34 – − 0.02	**0.032**	− 0.16	− 0.33 – − 0.00	**0.049**	− 0.21	− 0.36 – − 0.05	**0.009**
R^2^/R^2^ adjusted	0.214/0.208			0.171/0.166			0.143/0.137			0.219/0.213		

A total of 1,448 West German respondents were included in the analyses. The results of four different OLS-regressions are portrayed. Significant *p* values (< .05) are printed in bold

*std*. Standardized, *CI* 95% confidence interval, *GSI* Global Severity Index, *ACE* Adverse childhood experiences

## Discussion

The debate on adequate childcare diverged between the GDR and the FRG. In the West, maternal care was fostered and incentivized by the welfare state, whereas the GDR promoted female full-time employment. This article aimed to answer the questions of whether early external childcare is associated with mental health in adulthood and whether there are differences between those who were socialized in East or West Germany. To achieve this goal, group differences between childcare groups and between regions were tested using ANOVAs and ANCOVAs. Furthermore, OLS-regressions were estimated. Childcare groups could be divided into three forms according to whether they were receiving familial care or their age of first-time entering external care. The respondents’ psychological burden was indicated by the BSI-18, which included depression, anxiety, somatization, and their sum.

Although multiple claims and observations indicate the detrimental effect of early external childcare on children’s attachment styles, distress levels, behavior, and health, other evidence contradicts such associations. The results of this study suggest a similarly ambiguous tendency. External childcare was differently associated with the psychological burden of adults depending on socialization and current location in either East or West Germany. Notably, external care below the age of three exhibited significantly different associations with psychological burden between East and West Germans. However, only by tendency, it was associated with greater psychological burden for West Germans, and no relationship was found for East Germans in this childcare group. Compared to West Germans in familial care, tendencies of greater psychological burden were found for West Germans who had received external childcare before the age of three. Despite their marginal significance, the standardized β values which described the association between external childcare before the age of three and psychological burden in West Germany were higher than most of the control variables’ coefficients. Again, the results need to be interpreted with caution due to the rather small number of West German participants who entered external care before the age of three. Moreover, the dataset signa further information on the childhood of the participants (i.e., the socioeconomic status of their parents, the quality of the childcare facility they attended, exact age of entry into external care, exact time spent with caregivers, relationship quality with caregivers). Nevertheless, different implications can be discussed. A meta-analysis revealed that attachment behaviors, mother-child interactions, well-being of the child, the child’s social interactions with peers or nonparental adults, as well as the child’s cognitive development do not differ between children in maternal and nonmaternal care [40]. Moreover, as several studies confirmed that the bond between mothers and children does not suffer from maternal employment or external childcare [11, 12, 17, 18, 40, 41], influences other than extrafamilial care may be responsible for the greater psychological burden among West Germans who entered external care before the age of three. First, normalization of external childcare might be crucial in regard to the outcomes for children. As previous research has shown, the mother’s positive attitude toward external daycare as well as her commitment to work benefits the secure bond between her and her child [11, 12]. Another finding suggests that children profit if their mother’s attitude toward employment during her child’s infancy is consistent with her employment status [42]. Therefore, if external care is normalized, the likelihood that her attitude is more positive increases. As explained in the introduction, the East normalized early external childcare, whereas it has been distinctly less frequent in the West. Normalization might thus be one reason why East Germans did not exhibit associations between external care and psychological burden, but West Germans did. This assumption could be tested by differentiating between birth cohorts. Both in East and West Germany, external childcare has been normalized at some point which could also reflect in the psychological burden of the respective birth cohorts. Furthermore, the quality of childcare has improved over time, not least because experience and research led to improved childcare facility standards [24]. Second, a selection effect could explain the regional difference. Previous research has shown that in the past, poor households could only afford lower quality external care [43]. In contrast, before unification, the GDR paid for and normalized external care [2] which should decrease the probability of a selection effect for East Germans. External care of low quality as well as an economically disadvantaged household itself are related to greater psychological burden and its predictors [44, 45]. Thus, a possible explanation for the higher levels of psychological burden of those who entered external care before the age of three in the West lies in the lower socioeconomic status of their parent’s household which led to an external facility of low quality. However, West German children who attended external childcare below the age of three also were more likely to have more educated mothers which contradicts this assumption for some of the respondents. Since single mothers were more likely to rely on external care and because children of single mothers also report lower life satisfaction in adulthood as compared to those with two parents [46], the household constellation during childhood is another important predictor which should be acknowledged regarding our research question.

Future research should also focus on regional specifics regarding external care. Given that the sample size within this dataset was too small, long-term external care, which has been partially used for decades during times of the GDR, could not be integrated. Although the number of baby nurseries was formerly high in the FRG, as well, it drastically decrased until 1965, whereas the GDR recorded more than 5,000 places in baby nurseries in the same year [47]. However, long-term facilities, also accommodating children over weekdays with overnight stays or seasons, were found to inhibit children’s development; these facilities were reduced at a later point during the German division [24]. Due to the lower chances of secure attachment between children and caretakers within these long-term facilities, they should not be combined in analyses focusing on daycare centers. Associations between such forms of external care and later psychological burden should be observed. New policies should also be considered with larger sample sizes as they differed regionally. With this, potential cohort differences could be reflected.

Future research could also take a closer look at the conditions of the children’s homes. External childcare was found to be a protective factor for children from families that are related to violence or neglect: center-based care diminished the positive impact of children’s exposure to partner violence on their later behavioral outcomes [48]. With larger samples, an interaction term between adverse childhood experiences and the childcare group could be implemented to predict the potentially buffering effect of external care on psychological burden in adulthood.

Our findings suggest that external childcare below the age of three itself is not detrimental in the long term. We deduce this from the findings that there was no notable association between external childcare and psychological burden among East Germans. That there is a marginal significance of higher values of psychological burden for West Germans who attended external care below the age of three should thus not originate from the external care itself, but rather from the reason that lies behind deviating from such a widespread norm as familial care in West Germany. This is not only important to parents, but also to policymakers. To this day, full-time attendance in childcare facilities of toddlers is debated in West Germany. As a consequence, especially mothers reduce their work hours to care for children though society considers longer working hours ideal [49]. With an advanced normalization of external childcare, independent on the child’s age or the duration spent in daycare, the focus would shift to what is mostly influential which is the quality of parenting [50]. Therefore, it might be considered that the government subsidizes childcare costs for all children – at the times of writing, most German federal states subsidize the costs of childcare starting at the age of three. This way, families and countries would benefit financially from parents being able to work longer hours. Moreover, as is often demanded, families especially report that there is no place available for their child below the age of three [51].

Some limitations need to be mentioned. Due to the cross-sectional data, no causal links between early childcare and psychological burden in adulthood can be guaranteed. Moreover, the sample was reduced to those who were living in the broad region in which they grew up, suggesting that a selection effect of the remaining sample could not be ruled out. The variables related to the households in which the respondents grew up were not included in the dataset. However, previous research has shown that the quality of external childcare was lower among children from poorer households [43]. The socioeconomic status of the respondents’ upbringing should be an important confounder of the association between external childcare and psychological burden in adulthood. In general, attributes of external care should be beneficial for analyses but were not assessed. Only daily time spent in the external care facility was surveyed, however, this additional stratification yielded too small groups for analyses. While previous research has shown that daily time spent in external care is not related to psychological burden of East Germans [26], there may be differences among West Germans. Furthermore, the questions on childcare were answered retrospectively and subjectively which means that some respondents might have remembered it incorrectly or not at all. Additionally, the power of the analyses within this article was limited due to the small sample size of East Germans and partially small numbers of subgroups such as the number of West Germans who entered external care before the age of three. Since respondents were predominantly surveyed during the first spread of COVID-19 and the first lockdown in Germany, it was important to include the time of interviewing in the analyses. However, we could not control for the current pandemic situation in one’s area. In our analyses, respondents reported significantly higher psychological strain in February as compared to March or April 2020. Previous research also found some positive effects of lockdowns on psychological strain and distress which may be associated to the possibility to slow down their lives [52].

## Conclusions

External care below the age of three tended to be associated with higher levels of depression, anxiety, and global psychological burden among West Germans. Additionally, the relationship between psychological burden and the form of early childcare was significantly stronger among West Germans compared to the respective groups among East Germans. Extrafamilial care in daycare facilities was not related to the psychological burden of East Germans. Future research should observe whether selection effects, differing quality in childcare institutions, or differing norms are responsible for this disparity between regions.

## Data Availability

The datasets used and analyzed during the current study are available from the corresponding author on reasonable request.

## Abbreviations

ACE: Adverse Childhood Experiences
ANCOVA: analysis of covariance
ANOVA: analysis of variance
BSI-18: Brief Symptom Inventory-18
FRG: Federal Republic of Germany
GDR: German Democratic Republic
GSI: Global Severity Index
OLS: Ordinary least squares
Tukey’s HSD: Tukey’s honestly significant difference
